# Proton range verification in inhomogeneous tissue: Treatment planning system vs. measurement vs. Monte Carlo simulation

**DOI:** 10.1371/journal.pone.0193904

**Published:** 2018-03-05

**Authors:** Dae-Hyun Kim, Sungkoo Cho, Kwanghyun Jo, EunHyuk Shin, Chae-Seon Hong, Youngyih Han, Tae-Suk Suh, Do Hoon Lim, Doo Ho Choi

**Affiliations:** 1 Department of Radiation Oncology, Samsung Medical Center, Sungkyunkwan University School of Medicine, Seoul, Republic of Korea; 2 Department of Biomedical Engineering and Research Institute of Biomedical Engineering, College of Medicine, The Catholic University of Korea, Seoul, Republic of Korea; UNITED STATES

## Abstract

In particle radiotherapy, range uncertainty is an important issue that needs to be overcome. Because high-dose conformality can be achieved using a particle beam, a small uncertainty can affect tumor control or cause normal-tissue complications. From this perspective, the treatment planning system (TPS) must be accurate. However, there is a well-known inaccuracy regarding dose computation in heterogeneous media. This means that verifying the uncertainty level is one of the prerequisites for TPS commissioning. We evaluated the range accuracy of the dose computation algorithm implemented in a commercial TPS, and Monte Carlo (MC) simulation against measurement using a CT calibration phantom. A treatment plan was produced for eight different materials plugged into a phantom, and two-dimensional doses were measured using a chamber array. The measurement setup and beam delivery were simulated by MC code. For an infinite solid water phantom, the gamma passing rate between the measurement and TPS was 97.7%, and that between the measurement and MC was 96.5%. However, gamma passing rates between the measurement and TPS were 49.4% for the lung and 67.8% for bone, and between the measurement and MC were 85.6% for the lung and 100.0% for bone tissue. For adipose, breast, brain, liver, and bone mineral, the gamma passing rates computed by TPS were 91.7%, 90.6%, 81.7%, 85.6%, and 85.6%, respectively. The gamma passing rates for MC for adipose, breast, brain, liver, and bone mineral were 100.0%, 97.2%, 95.0%, 98.9%, and 97.8%, respectively. In conclusion, the described procedure successfully evaluated the allowable range uncertainty for TPS commissioning. The TPS dose calculation is inefficient in heterogeneous media with large differences in density, such as lung or bone tissue. Therefore, the limitations of TPS in heterogeneous media should be understood and applied in clinical practice.

## Introduction

Proton therapy is a recently developed state-of-the-art radiation therapy technique. It has the advantage of delivering a minimum dose to normal organs, while delivering a large dose to cancer cells, using a physical property known as the Bragg peak of the proton [1–3]. A dose to normal tissue proximal to the target can be greatly reduced by accurately positioning the Bragg peaks at the target. This is done by controlling the range of the proton beams. The Samsung Medical Center Proton Therapy Center (SMC-PTC) is the first private hospital-based proton therapy center in Korea [4]. SMC-PTC treated its first patient in December 2015, and currently treats cancer patients with both wobbling and line-scanning proton therapy modes.

In order to treat cancer patients, it is necessary to predict the dose distributions in a patient using dose calculation algorithms. The human body is a heterogeneous medium that includes many organs with different atomic numbers and densities. Therefore, different levels of calculation algorithms have been developed for the clinical use of proton beams, such as the pencil beam (PB) algorithm [5–7] and Monte Carlo (MC) simulations [8–11]. In order to use algorithms in clinical practice, high accuracy in dose calculation and short calculation times are required. MC simulations provide the most accurate dose predictions [12]. By simulating the radiation transport and scoring energy deposition, it can accurately account for proton number and electron density variations within a patient. However, MC simulations require very long computation times because of the stochastic characteristics of dose calculation. This disadvantage limits clinical applications, and there is no commercial treatment planning system (TPS) based on MC simulations for proton therapy yet.

The majority of proton therapy centers use a commercial TPS that utilizes PB algorithms for dose calculation of passively scattered and scanning proton therapy. The PB algorithm features a short calculation time, and dose calculation accuracy in a homogeneous medium is satisfactory within clinical criteria. However, in heterogeneous media, the multiple Coulomb scattering (MCS) used in the PB algorithm can be implemented only along the beam axis [13–15]. Sawachchi et. al., reported that the PB algorithm may have an error of 2 mm at the distal fall-off Bragg peaks due to the limitation of range calculation, resulting from imperfect MCS modeling with the PB algorithm in heterogeneous media. Therefore, verifying the range calculation in heterogeneity in clinically relevant conditions and assessing the inherent error associated with the algorithm is necessary when commissioning a TPS with the PB algorithm.

Many studies have assessed PB algorithm range uncertainty by comparing it to MC simulation. For MC simulations to be reliable, they must be commissioned against the measurement. However, measuring ranges in tissue inhomogeneity is a difficult task because of the non-solid nature of organs, and involves a large setup and dosimetry uncertainty. The purpose of this study is to investigate the accuracy of proton ranges in heterogeneities computed by the PB algorithm implemented in a commercial TPS and by MC simulation against measurement. For that purposes, we used a computed tomography (CT) calibration phantom that has inhomogeneity plugs with a 25-mm diameter installed. The ranges from TPS and MC simulation were quantitatively assessed, and the range uncertainty of the PB algorithm implemented in a commercial TPS was investigated.

## Materials and methods

### 1. Treatment plan

**Fig 1:**
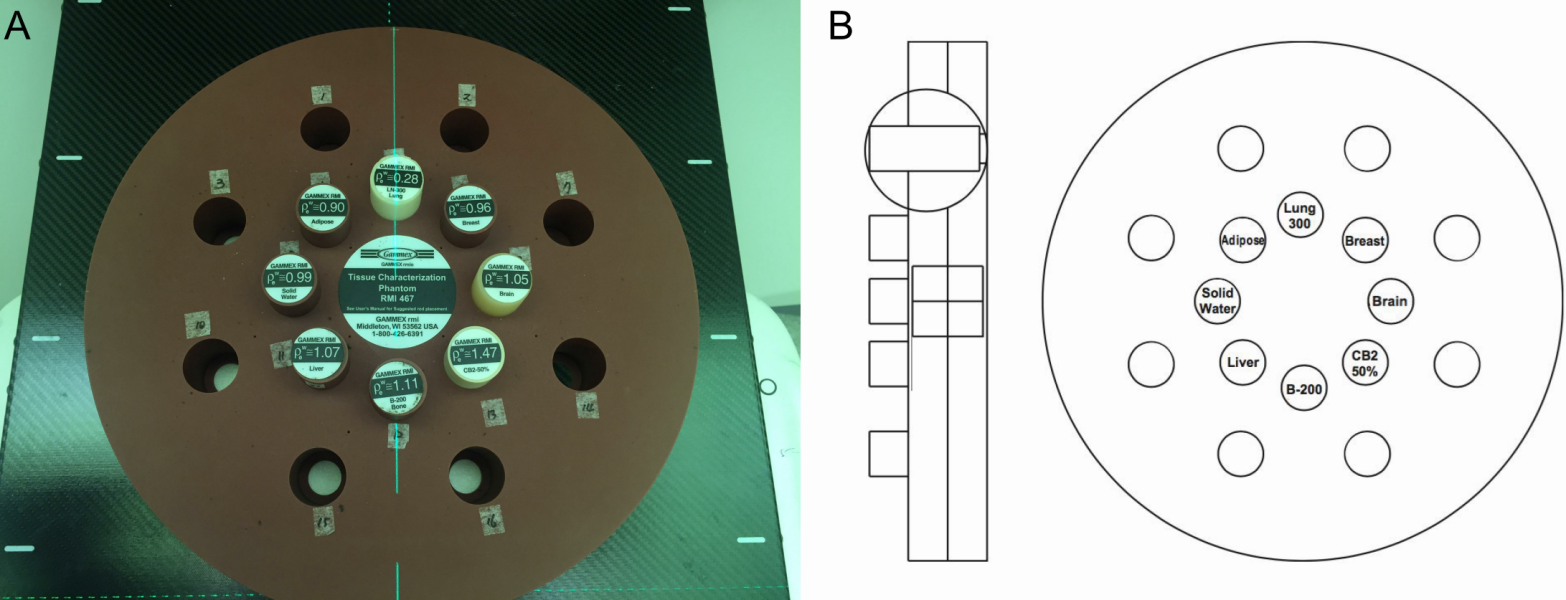
Gammex 467 tissue characterization phantom. A: Photographs of the Gammex phantom with eight different inhomogeneity rods inserted in the inner circle. B: Layout of the Gammex phantom.

To evaluate the dose calculation algorithm, a Gammex 467 Tissue Characterization Phantom (Gammex Inc., Middleton, WI, USA) was used. CT images were acquired using a Discovery CT590 RT (GE Healthcare, Milwaukee, WI, USA) with a 120 kV X-ray tube voltage. The planning volume was scanned with a 2.5 mm slice thicknesses. Eight different tissue-equivalent material cylinder rods, 25 mm in diameter and 70 mm long, were plugged into the phantom. The phantom was laid down on the couch with the rods parallel to the transverse CT imaging plane. Fig 1 shows the setting geometry of the Gammex phantom used in this study. The inhomogeneity material characteristics were taken from ICRU Reports 44 [16] and 46 [17]. The tissue compositions and physical densities are listed in Table 1.

**Table 1:** Chemical composition of the eight materials used in this study.

Rod Materials	Density (g/cm^3^)	Elemental Weights in Percentage Points (%)											
		H	C	N	O	Na	Mg	P	S	Cl	K	Ca	Fe
LN-300 Lung	0.3	0.103	0.105	0.031	0.749	0.002		0.002	0.003	0.002	0.003		
Adipose	0.94	0.114	0.598	0.007	0.278	0.001			0.001	0.001			
Breast	0.98	0.109	0.506	0.023	0.358	0.001		0.001	0.001	0.001			
Solid Water	1.02	0.112			0.888								
Brain	1.05	0.102	0.143	0.034	0.71	0.001		0.002	0.003	0.001	0.004		
Liver	1.1	0.102	0.139	0.03	0.716	0.002		0.003	0.003	0.002	0.003		
B-200 Bone Mineral	1.15	0.085	0.404	0.058	0.367	0.001	0.001	0.034	0.002	0.002	0.001	0.044	0.001
CB2-50%	1.56	0.056	0.235	0.05	0.434	0.001	0.001	0.072	0.003	0.001	0.001	0.146	

CB2-50%: Calcium carbonate bone

A wobbling proton treatment plan was generated for the phantom CT images using a RayStation (Version 5.0.1, RaySearch Medical Laboratories AB, Stockholm, Sweden), which calculated the dose with a PB algorithm. One wobbling-mode anterior proton beam was planned to pass through all inhomogeneity rods, with 5 cm of spread-out Bragg peaks (SOBP). In order to evaluate the proton beam percent depth dose (PDD) that passed through the Gammex phantom, we inserted a virtual solid water phantom downstream of the Gammex phantom. The solid water phantom (Sumitomo Heavy Industries Ltd., Tokyo, Japan) was made of high-density polyethylene, and the density of this phantom was 0.95 g/cm^3^. The chemical compositions were 14.3% hydrogen and 85.6% carbon. The composition of the virtual water is different from water, and the mean excitation energy of the solid water phantom was 57.5 eV, which is different from that of 75 eV for water [18–20]. However, the Hounsfield unit (HU) of the solid water phantom was quite similar to that of real water when it was scanned with CT. Therefore, we used the virtual solid water phantom, which was assigned a density of 0.95 g/cm^3^ in the TPS with correct chemical composition, instead of the scanned solid water phantom CT images. The beam isocenter was located at the geometric center of the Gammex phantom, and the proton field had a circular shape, with a 15-cm diameter collimated by a brass aperture, which was wide enough to irradiate all rods in the phantom. The proton energy was set to 190 MeV without using a range compensator, so that the distal fall-off of SOBP was located at the virtual phantom. In the proton therapy system in the SMC-PTC, SOBPs were created with ridge filters for widths from 1 cm to 16 cm. For this study, we selected a ridge filter of 5 cm to generate an SOBP with 5 cm width with 19.7 cm range, using the wobbling proton beam. The voxel size for all dose calculations was set to 2 mm.

### 2. Monte Carlo simulation

**Fig 2:**
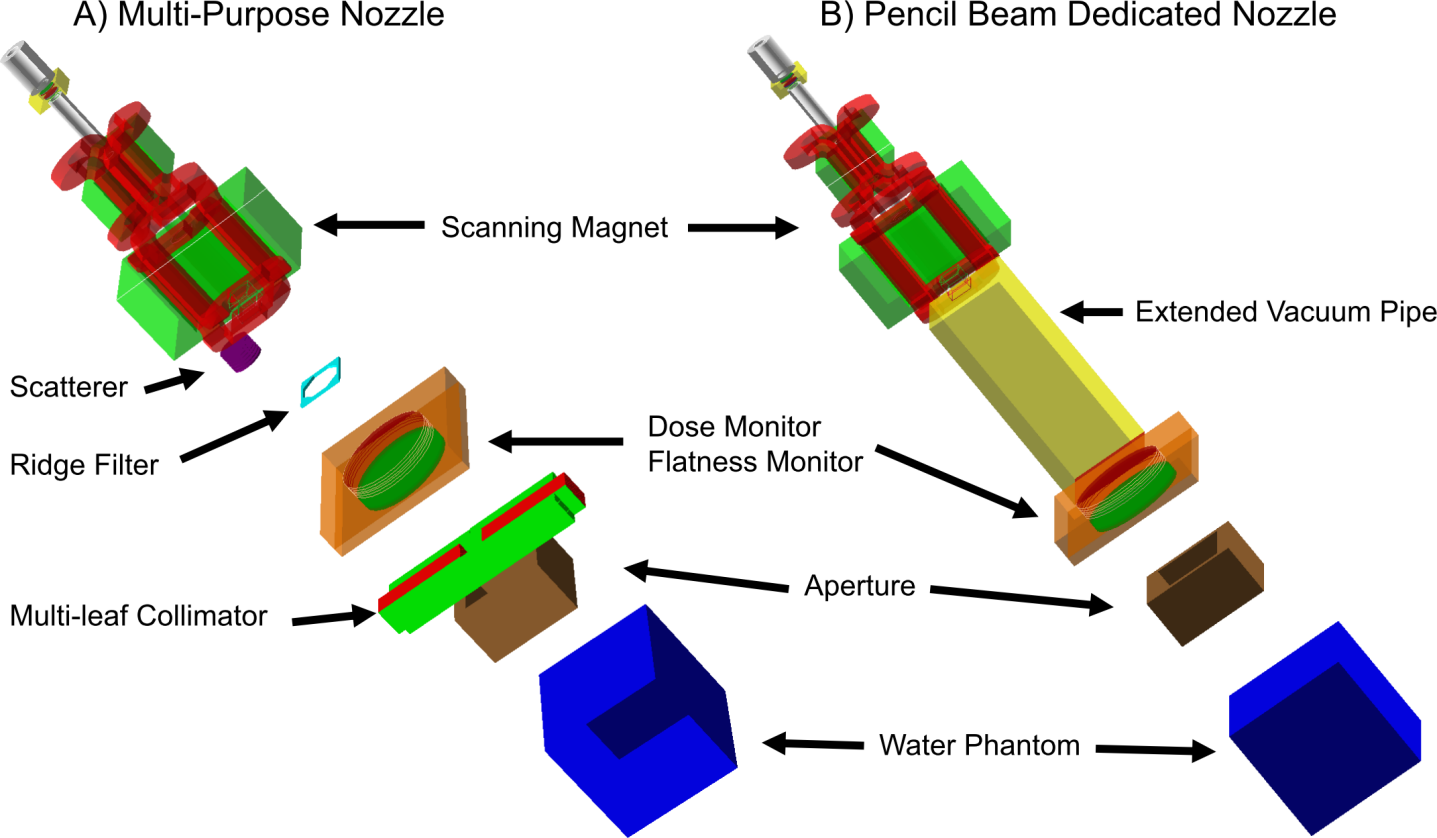
3-D volume rendering of the nozzle modeled in GEANT4. A: A multi-purpose nozzle consisting of a wobbling magnet, scatterer, ridge filter, multi-leaf collimator, and snout; B: Pencil beam dedicated nozzle consisting of scanning magnet, extended vacuum pipe, and snout.

**Fig 3:**
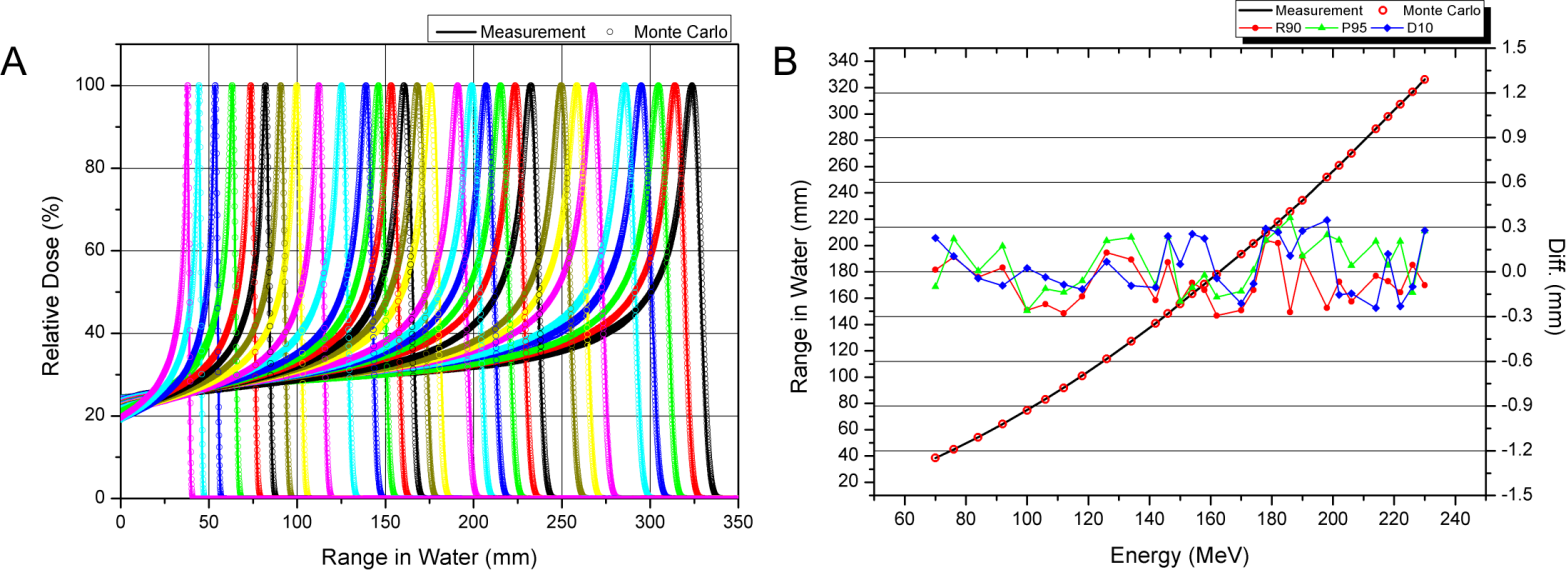
Validation of Monte Carlo simulation compared with measurement. A: PDDs for 30 different ranges of pristine Bragg peak from 70 MeV to 230 MeV, B: Comparison of measured range (solid black line) and simulated range (open red circle) in water according to proton energy. Distal 90% range (red), proximal 95% range (green), and distal 10% range (blue).

MC simulations were performed with the GEANT4 toolkit (Version 10.01.p01). This is one of the most widely used toolkits for proton therapy dose calculations [21]. GEANT4 is free, open-source code written in C++, and is widely used in high-energy physics and space physics. Many studies on proton therapy have used GEANT4 to calculate accurate proton doses for the complex human body [22–24]. For simulation, we created a virtual machine by modeling all the components in the proton nozzle installed at SMC-PTC. These included models for the scatterers, wobbling magnets, ridge filters, profile monitors, snout, aperture, compensator, and multi-leaf collimator, based on the information provided by the manufacturer. The developed MC simulation code was confined to the exit of the beam transport system at the end of the target phantom under the assumption that the entering proton beam encounters the first element of the nozzle with a Gaussian energy distribution, and is parallel to the central axis of the scanning magnet of the nozzle. Although only the wobbling mode of a multi-purpose nozzle was used in this study, the MC code was developed for both the multi-purpose nozzle, which provides wobbling and scanning treatment modes, and the scanning dedicated nozzle. The two implemented types of proton treatment nozzles are presented in Fig 2. The developed MC simulation code was first validated against the measured pristine Bragg peak data taken for commissioning the TPS. As shown in Fig 3, we compared the PDDs of the pristine Bragg peak computed by the MC simulation, and those for the measured data for 30 different energies. At all 30 energies, the distal 90% were within 0.3 mm, and the largest error was -0.29 mm at 162 MeV. All differences in the proximal 95% distance were within 0.4 mm, and the largest error was 0.36 mm at 186 MeV. Excellent agreement was found between the two data series for the distal range, plateau dose, and distal edge.

In this study, MC simulation was performed in two steps. First, a wobbling proton beam with 190 MeV energy and 5 cm wide SOBP was produced and passed through the proton nozzle. The particle types, energies, positions, and angular momentums values were stored in the phase-space file at the end of the proton nozzle. A total of 10^9^ histories were simulated to ensure that the statistical uncertainty was less than 1% for all dose points. The cut-off range for all particles was set to 0.01 mm. Thus, when the energy of the particle was less than the cut-off range value, all the primary and secondary particles were locally deposited. The second step was to model a Gammex phantom located at the isocenter with eight different material rods inserted. The density and atomic composition of each material summarized in Table 2 were assigned to each type of rod. A solid water phantom was placed downstream from the Gammex phantom, and the PDDs were calculated along the line passing through each rod. The sensitive detector volume in the solid water phantom was subdivided into voxels of 1×1×1 mm^3^. All MC simulations were performed on a Linux computer with 104 Intel Xeon E5-2690 v2 3.0 GHz CPUs and 40 GB RAM.

**Table 2:** Range (R90) and gamma pass rate of measurement, MC simulation and TPS for reference data in an infinite solid water phantom and eight materials in the Gammex phantom.

Material	Mea.	Monte Carlo					TPS			
	Range (mm)	Range (mm)	Diff. (mm)	Gamma passing rate (Max. Gamma Index)		Range (mm)		Diff. (mm)	Gamma passing rate (Max. Gamma Index)	
				No supp.	10% supp.				No supp.	10% supp.
Reference	171.3	171.7	0.4	96.5 (1.2)	96.0 (1.2)	171.8		0.5	97.7 (1.2)	97.3 (1.2)
LN300	198.1	196.6	-1.5	85.6 (1.3)	85.1 (1.4)	176.3		-21.8	49.4 (4.5)	48.0 (4.5)
Adipose	152.4	152.7	0.3	100.0 (1.2)	100.0 (1.0)	154.2		1.8	91.7 (2.6)	99.2 (1.1)
Breast	151.1	150.5	-0.6	97.2 (1.2)	97.7 (1.2)	150.7		-0.4	90.6 (3.5)	100.0 (0.9)
Solid Water	149.1	150.2	1.1	92.8 (1.3)	100.0 (0.7)	149.4		0.3	90.6 (3.3)	100.0 (0.8)
Brain	146.7	146.2	-0.5	95.0 (1.1)	100.0 (0.7)	149.5		2.8	81.7 (4.6)	91.1 (1.3)
Liver	143.2	142.9	-0.3	98.9 (1.0)	98.3 (1.0)	144.9		1.7	85.6 (3.6)	98.3 (1.1)
Bone mineral	141.5	140.9	-0.6	97.8 (1.0)	100.0 (1.0)	141.7		0.2	85.6 (3.6)	100.0 (0.7)
CB2-50%	119.9	118.4	-1.5	100.0 (0.7)	100.0 (0.7)	122.5		2.6	67.8 (3.4)	87.8 (1.3)

CB2-50%: Calcium carbonate bone

supp.: suppressing dose

### 3. Measurement

Among the two nozzles, a measurement was made in a multi-purpose nozzle with a wobbling beam of 190 MeV with a 5-cm ridge filter. Three categories of wobbling beam, according to field size, are available: small, medium, and large wobbling radius. The middle wobbling radius was selected for a 15-cm block diameter.

Reference data were obtained to verify the MC simulations and TPS calculations by measuring the rod-free area in the center of the Gammex phantom, where the proton beam passed through the 5-cm depth of the solid water material. A ZEBRA (IBA-Dosimetry, Schwarzenbruck, Germany) was used to measure the central axis of the beam. The PDDs of the eight different materials were measured using an OCTAVIUS Detector 729 Chamber Array (PTW, Freiburg, Germany) because the dose measurement passing through the inhomogeneity plug was off-axis and not feasible with ZEBRA. Hence, the two-dimensional absolute dose was measured by changing the thickness of the solid water phantom downstream of the Gammex phantom, with 1 mm intervals. After all measurements were completed, we constructed the PDD with a point dose at 1 mm depth increments along the central position of each rod.

### 4. Evaluation method

For the evaluation of two different PDDs, we performed a one-dimensional gamma index analysis, which is a well-established method for quantitatively comparing dose distributions [25]. For gamma analysis, 3 mm/3% of agreement with low dose suppression was conducted, which is the same criterion and method used in clinical plan evaluation, and re-analysis was performed without suppressing the low 10% of the maximum dose, to analyze the full range of PDD. We used the global gamma criterion by selecting 3% of the maximum plan dose rather than the local dose at the comparison point.

## Results and discussion

The goal of this study was to investigate the dose calculation accuracy of proton therapy by TPS in heterogeneous media compared to measurement using a CT calibration phantom.

**Fig 4:**
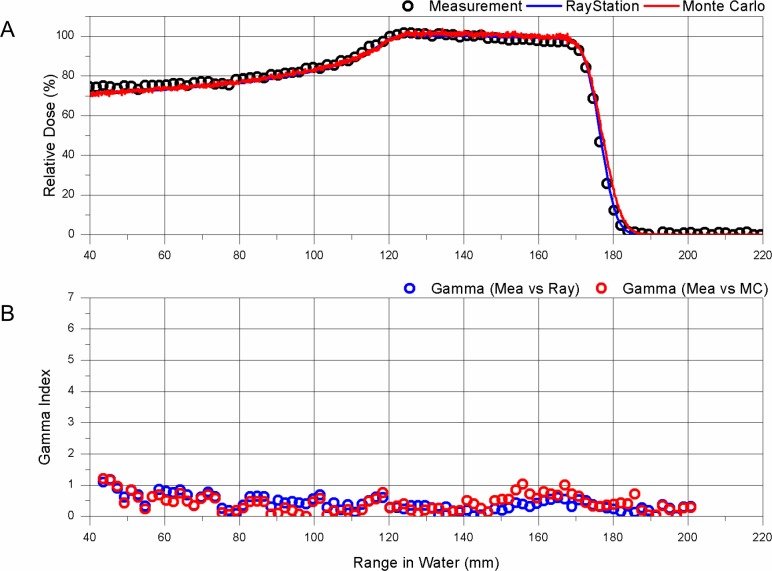
PDD comparison for infinite solid water. A: Measurement (open black circle), TPS (solid blue line), MC (solid red line); B: 1D gamma evaluation between measurement and TPS (open blue circle), and between measurement and MC (open red circle).

**Fig 5:**
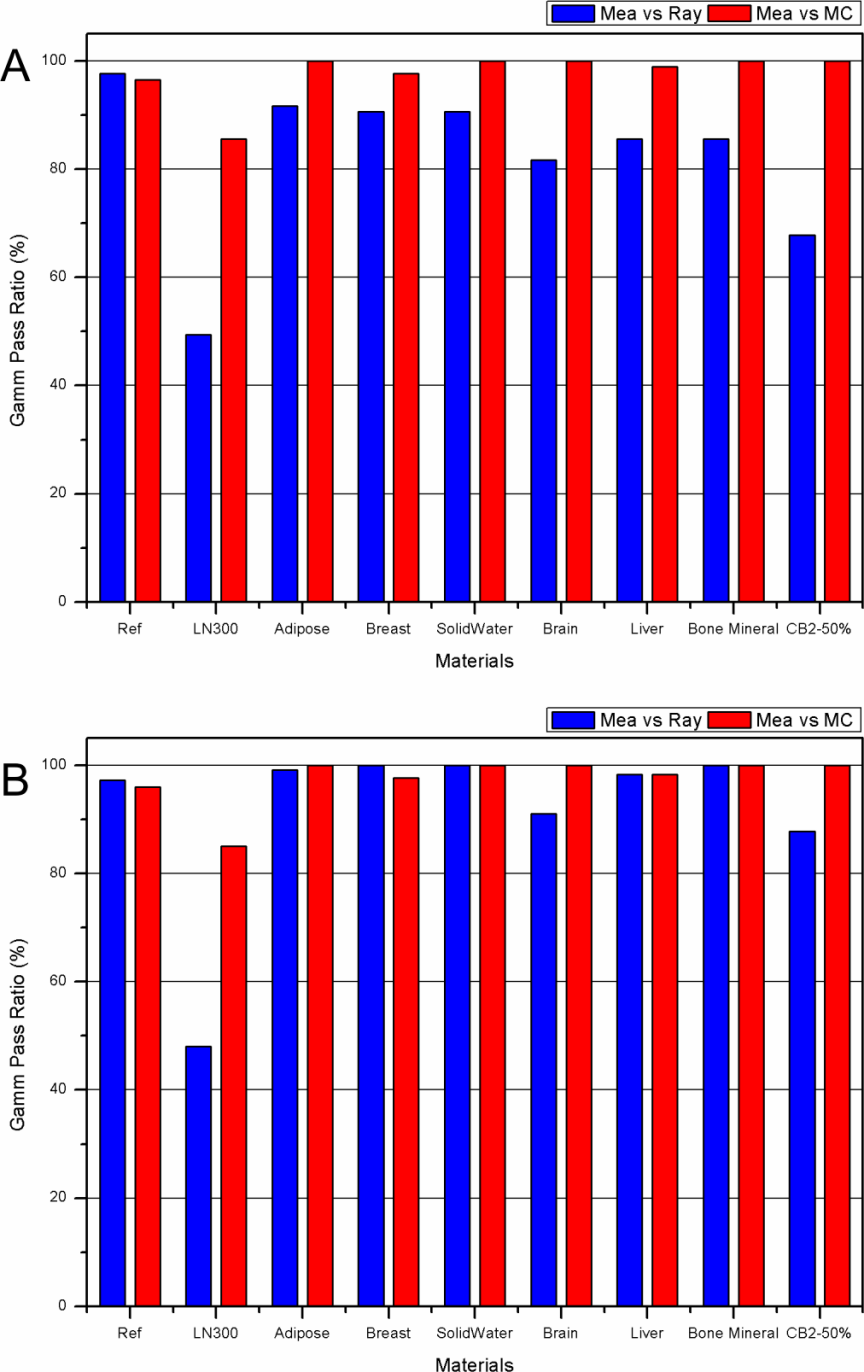
**Histogram for 1D gamma passing rate for eight materials: (A) without low dose suppressing and (B) with suppressing of 10% of the maximum dose.** Comparison with measurement and TPS (blue bar); comparison with measurement and MC (red bar).

**Fig 6:**
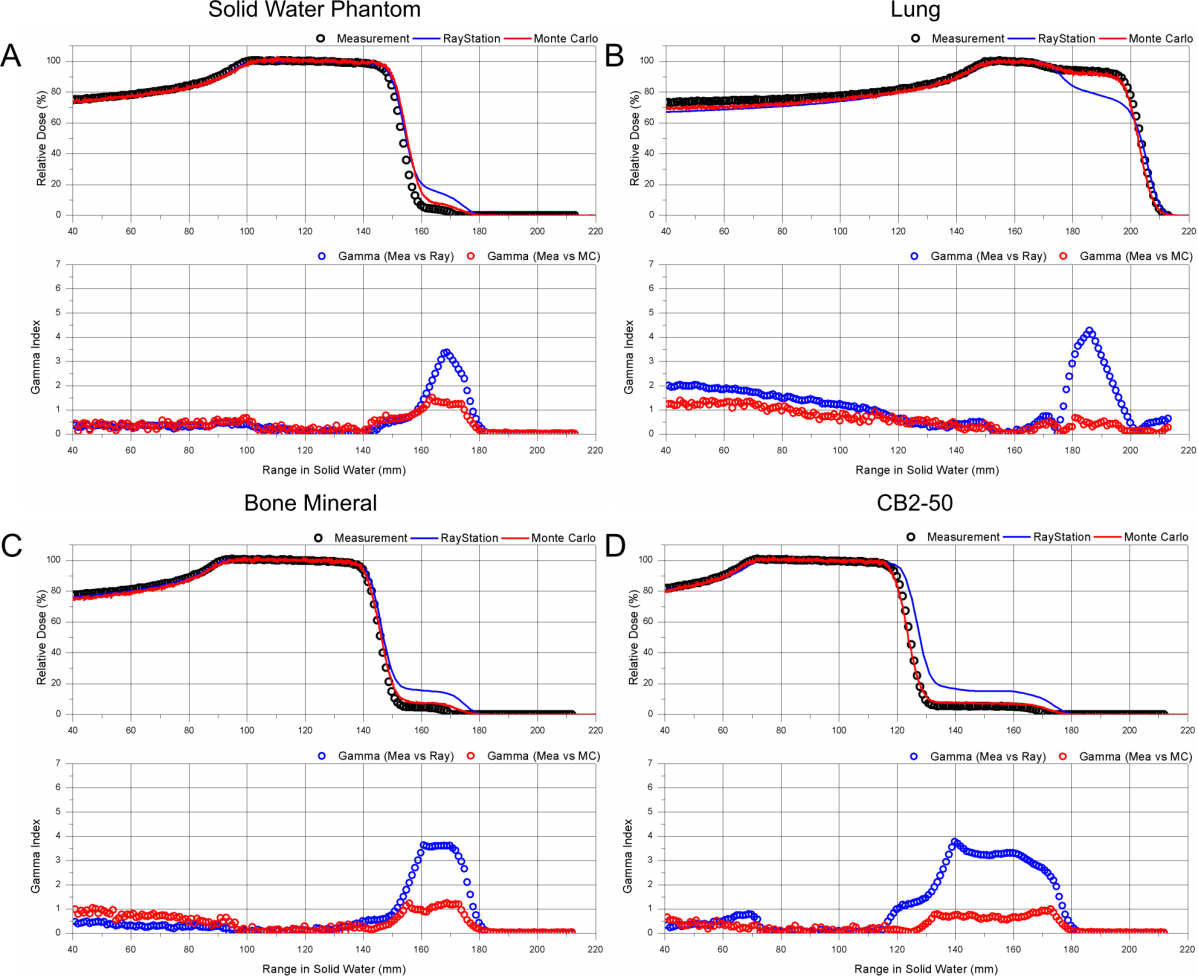
PDD comparison for tissue equivalent materials. A: Solid water, B: lung, C: bone mineral, D: calcium carbonate bone; PDD: Measurement (open black circle), TPS (solid blue line), MC (solid red line); 1D gamma evaluation between measurement and TPS (open blue circle), and between measurement and MC (open red circle).

The measured and the calculated PDD of the proton beam, as reference data in the solid water phantom, along the central axis, are presented in Fig 4. The distal range of 90% and SOBP width were 171.3 mm and 55.0 mm for measurement, 171.8 mm and 54.7 mm for TPS, and 171.7 mm and 53.7 mm for MC simulation, respectively. The gamma passing rates for all ranges of PDD between measurement and TPS calculations were above 97.7%, and between measurement and MC simulation were 96.5% (Fig 5). Although some points in the gamma index of the TPS at the entrance and the distal fall-off were greater than 1, TPS calculation and MC simulation prediction showed good agreement with measurements when the medium was infinitely large. The observed difference in the entrance region was partially due to the measurement uncertainty and limitations of the calculation algorithm. However, when the proton beam passed through the solid water rods, which were 25 mm in diameter and 70 mm in length, located off-axis from the beam, different PDDs can be seen, as shown in Fig 6. The distal range of 90% of PDD of the proton beam passing solid water that was 2 cm thicker than the central axis’s solid water of Gammaxphantom, was 149.1 mm for measurement, 149.4 mm for TPS, and 150.2 mm for MC simulation. However, the dose distribution of the distal tail region was different from that of the central axis, as shown in Fig 6, where a higher dose at the 10% level of the maximum dose was observed. The distal range of the 10% dose was 159.3 mm for measurement, 171.7 mm for TPS, and 162.7 mm for MC simulation. The TPS overestimated the distal low dose, potentially because the TPS PB algorithm does not accurately reflect the lateral scattering of the interface between the solid water rod and air. This phenomenon was also observed in the PDDs of beams passing through rods of other materials.

The PDD of lung tissue with a density of 0.30 g/cm^3^ is presented in Fig 6B. The distal range of 90% of the MC simulation and measurement differed by -1.5 mm. The gamma passing rate was 85.6%, and the gamma index was above 1 at the entrance region. The result of MC simulation agreed with measurement within 3% of the differences within the SOBP region. In the case of TPS prediction, the dose in the distal region of the SOBP collapsed more than 20%. The largest gamma index was approximately 4.5 near the distal shoulder of the SOBP and the gamma passing rate was 49.4%, indicating that the TPS calculation was not accurate. In TPS, the MCS calculation does not consider lateral inhomogeneities. Larger errors occurred because the rod size of the lung material used in this experiment was small. However, the MC that reflects the MCS in inhomogeneities had a high calculation accuracy.

The PDD for bone mineral with a density of 1.15 g/cm^3^ and a calcium carbonate bone with a density of 1.56 g/cm^3^ are presented in Fig 6C and 6D, respectively. In the case of bone mineral, the distal range of 90% shows good agreement among the measurement, TPS, and MC. The gamma passing rate was 97.8% between measurement and MC simulation and 85.6% between measurement and TPS, without suppressing the low dose. While suppressing the low dose, the gamma passing rate for both of TPS and MC simulation increased to 100%. The MC simulation for bone mineral was found to agree with the measured data within the 3 mm/3% criteria, although the gamma index was slightly over 1.0 in the distal SOBP region. In addition, the distal tail dose lower than 10% of the maximum dose agreed with the measurement with an error of 3%. However, in the case of TPS, in the distal tail region, for approximately 10% of the maximum dose, the gamma index was larger than 3.6, indicating that the TPS dose calculation overestimated the low-dose distal region. For the calcium carbonate bone material, the dose calculated with MC simulation and that with TPS showed larger differences. The range difference between the measurement and MC was -1.5 mm, and that between the measurement and TPS was 2.6 mm. The TPS calculation overestimated the dose in the distal tail region, similar to that with the bone mineral material.

**Fig 7:**
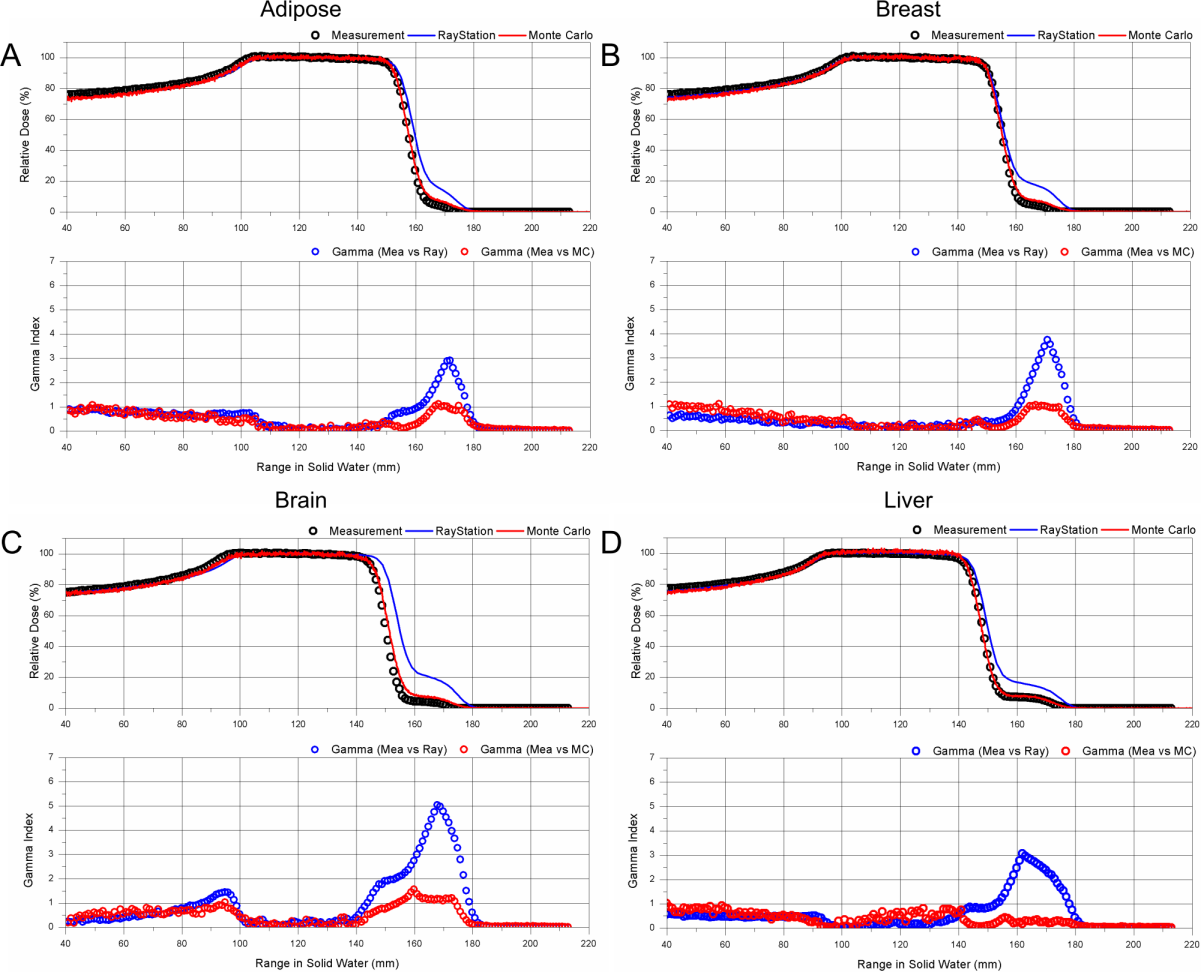
PDD comparison for tissue equivalent materials. A: Adipose, B: Breast, C: Brain, D: Liver; PDD: Measurement (open black circle), TPS (solid blue line), MC (solid red line); 1D gamma evaluation between measurement and TPS (open blue circle), and between measurement and MC (open red circle).

The ranges and gamma passing rate for other tissue-equivalent materials, such as adipose, breast, brain, and liver are presented in Table 2, together with those for lung, bone mineral, and calcium carbonate bone (CB2-50%). MC simulation results were in good agreement, within 1.5 mm of the measured data for all tissue equivalent materials. MC simulation accurately predicts the dose change in the distal tail region, caused by the interface of various materials as shown in Figs 6 and 7. The range from TPS calculation was rather different from the measurements. The brain tissue material showed the largest difference of 2.8 mm, in the distal range of 90%. Moreover, dose calculations by TPS near the distal tail region were inaccurate for all materials. The observed inaccuracy of the TPS prediction is potentially due to the central axis approximation that assumes that the material is infinite in the lateral direction, which is different from the experiments with inhomogeneity rod with a diameter of 25 mm. The dose calculation by TPS does not cause any large deviation when the substance has a simple geometry such as the reference case in this study, but a large deviation occurs when the structures have a large density difference along the beam.

These observed inaccuracies agree with the work by Hong et. al., [5] who reported that the PB algorithm produces errors in the shadow of thick inhomogeneities when the interface is parallel to the beam’s central axis. In addition, several studies found that MCS occurring within the heterogeneities is the primary contributor to distal edge degradation [14, 26, 27]. To overcome the limitations accompanied by the slab approximation in the PB algorithm, Szymanowski and Oelfke [13], and Egashira [28] suggested two-dimensional scaling of the lateral proton fluence. However, this new concept has not been implemented in a commercial TPS, to the best of our knowledge, because of the intensive computation processes that result in relatively larger computation times.

For successful patient treatment in clinical practice, the aforementioned range uncertainties in various clinical situations must be accurately taken into account in treatment planning. Paganetti [29] conducted a comprehensive study on the range margin associated with the planning process for both the analytical algorithm and the MC simulation. More specifically, Schuemann et. al., [30] recommended a site-specific margin because of the range uncertainty of the PB algorithm, by comparing clinically treated patient plans with MC calculations. These were 2.8% for liver and prostate, 3.1% for whole brain, 5.9% for lung and breast, and 6.5% for head and neck. However, the range uncertainty in the heterogeneous media must be investigated for each proton therapy center, because the characteristics of the equipment are different for each proton therapy system and are also dependent on the commissioning of the TPS. One specific lesson from our study results is that we must reconsider the range margin in the treatment of the lung tumor close to the chest wall. If the proton beam passes along the interface between the chest wall and lungs, which is a measurement condition in this study, the range uncertainty can be up to 10% for the distal 90% range, and the dose in the small area of SOBP can be 20% smaller than that from the TPS calculation, as summarized in Table 2. However, the MC-computed 90% of the maximum dose range agreed with measurements within -0.8% of the difference, although some difference in the SOBP in the lung (Fig 6B) and low-dose tail regions were observed in the adipose, breast, bone mineral, and calcium carbonate bone (Figs 6 and 7).

**Fig 8:**
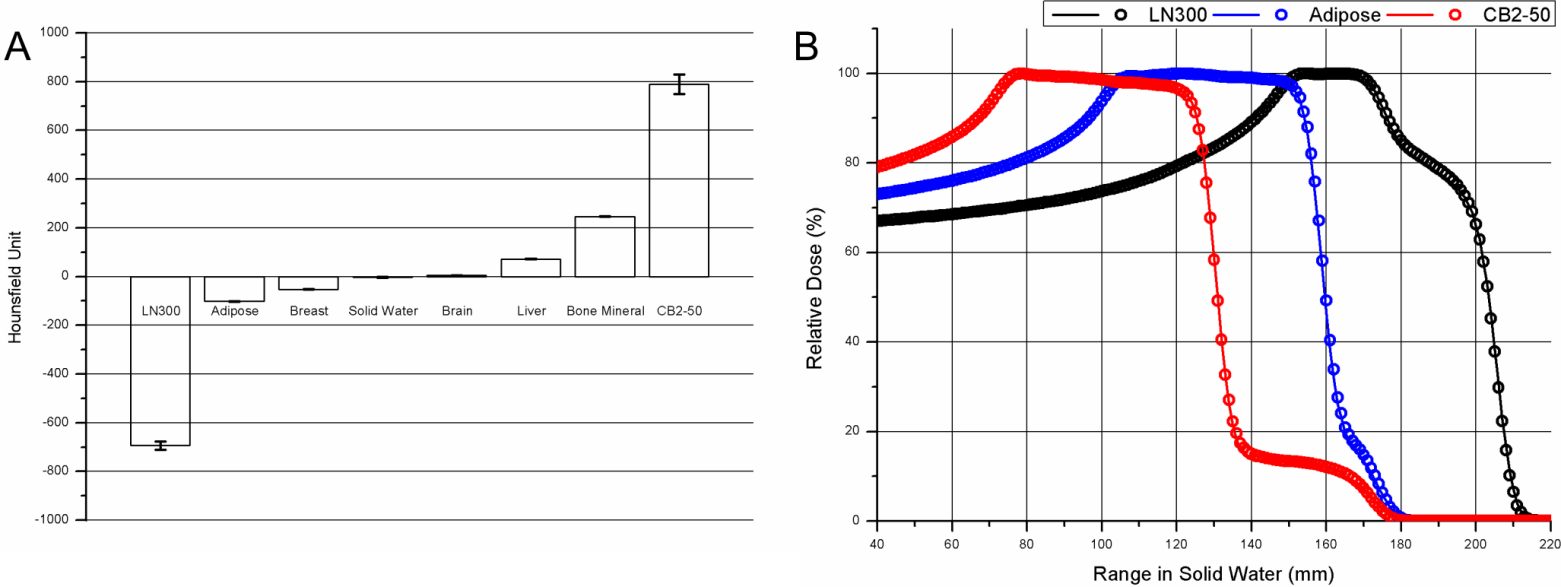
PDD according to Hounsfield unit. A: Histogram of HU for eight materials according to changes in their position. B: PDD based on the position change in three selected materials. Lung (black), adipose (blue), calcium carbonate (red).

The HU value changed for the variation in position of the phantom because of the difference in attenuation length. In this study, the positional variation does not affect the result, as shown in Fig 8. For a different CT image with changed rod positions, the HU value was analyzed, and the dose was calculated through TPS. The standard deviation of calcium carbonate bone showing the largest change in HU value was 41.0 HU, and that for the lung tissue showing the second largest change was 16.5 HU. The PDD according to the different rod position showed sub-millimeter range differences. The difference was 0.38 mm in lung tissue, 0.33 mm in adipose tissue, and 0.02 mm in calcium carbonate bone.

Complex causes affect the final deviation of dose distribution. These could include Hounsfield unit-stopping power (HU-SP) uncertainty, inaccuracy of the PB algorithm, poorly completed TPS commissioning, among other reasons. Our results indicate that there could be a large inaccuracy in lung tissue interface when slab approximation of PB algorithm was not satisfied. Another importance source of uncertainty is the HU-SP conversion uncertainty. The nonlinear HU-SP relation may resolve the problem [31] or one can re-optimize the HU-SP curve for the lung region by performing the proton radiography [32]. We could not improve the accuracy of the TPS computation; however, optimizing the HU-SP relation could be on option for improving the range accuracy.

## Conclusions

We could successfully assessthe accuracy of range computation for the PB algorithm in a TPS and MC simulation compared with measurements using a CT calibration phantom. As expected, there was a small difference in the proton range between measurement and calculation from TPS when infinitely homogeneous solid water is considered as the medium. However, the accuracy of dose computations by TPS was reduced with a finite volume for inhomogeneities as we tested with a complex geometry to calculate by TPS.

Consequently, when treating a target with complex geometry in inhomogeneous tissue, especially for lung tissue, the proton dose in the SOBP in the lung tissue can be smaller than the TPS computation, and the range can be shorter by up to 10%, which potentially affects the dose volume histogram prediction of the target and the nearby lung tissue. MC simulation can predict the range of the proton beam accurately, even though a low dose in the trail can be slightly overestimated. Validating the dose computation algorithm for the inhomogeneity poses the accuracy level of TPS, thereby users understand the plan robustness near the organs with density inhomogeneity.

Using a similar measurement design, the limitations of TPS calculation accuracy for line-scanning proton beams in heterogeneous media will be investigated in the near future.

## Supporting information

S1 File Raw data for reference PDD (spreadsheet file). (XLSX)

S2 File Raw data for eight different materials (spreadsheet file). (XLSX)

S3 File Raw data for gamma passing rate (spreadsheet file) https://figshare.com/s/9f131bc918941f126d88. (XLSX)
